# User Experiences With and Recommendations for Mobile Health Technology for Hypertensive Disorders of Pregnancy: Mixed Methods Study

**DOI:** 10.2196/17271

**Published:** 2020-08-04

**Authors:** Karin Rolanda Jongsma, Josephus F M van den Heuvel, Jasmijn Rake, Annelien L Bredenoord, Mireille N Bekker

**Affiliations:** 1 Department of Medical Humanities University Medical Center Utrecht Utrecht University Utrecht Netherlands; 2 Obstetrics and Gynaecology University Medical Center Utrecht Utrecht University Utrecht Netherlands

**Keywords:** mobile health, hypertension, telemonitoring, ethics, high-risk pregnancy, preeclampsia, digital health

## Abstract

**Background:**

Hypertensive disorders of pregnancy (HDP) are a primary cause of adverse maternal and neonatal outcomes worldwide. For women at risk of hypertensive complications, guidelines recommend frequent surveillance of blood pressure and signs of preeclampsia. Clinic visits range from every 2 weeks to several times a week. Given the wide ubiquity of smartphones and computers in most countries and a growing attention for self-management, digital technologies, including mobile health (mHealth), constitute a promising component of monitoring (self-measured) blood pressure during pregnancy. Currently, little is known about the experiences of women using such platforms and how mHealth can be aligned with their needs and preferences.

**Objective:**

The objectives were twofold: (1) to explore the experiences of Dutch women who had an increased risk of HDP with a blended care approach (mHealth combined with face-to-face care) for remote self-monitoring of blood pressure and preeclampsia symptoms and (2) to formulate recommendations for the use and integration of mHealth in clinical care.

**Methods:**

Alongside a prospective blended care study (SAFE@home study) that monitors pregnant women at increased risk of HPD with mHealth technology, a mixed methods study was conducted, including questionnaires (n=52) and interviews (n=11). Results were analyzed thematically.

**Results:**

Of the 4 themes, 2 themes were related to the technologies themselves (expectations, usability), and 2 themes were related to the interaction and use of mHealth (autonomy and responsibilities of patients, responsibilities of health care professionals). First, the digital platform met the expectations of patients, which contributed to user satisfaction. Second, the platform was considered user-friendly, and patients favored different moments and frequencies for measuring their blood pressure. Third, patient autonomy was mentioned in terms of increased insight about their own condition and being able to influence clinical decision making. Fourth, clinical expertise of health care professionals was considered essential to interpret the data, which translates to subsequent responsibilities for clinical management. Data from the questionnaires and interviews corresponded.

**Conclusions:**

Blended care using an mHealth tool to monitor blood pressure in pregnancy was positively evaluated by its users. Insights from participants led to 7 recommendations for designing and implementing similar interventions and to enhance future, morally sound use of digital technologies in clinical care.

## Introduction

Mobile health (mHealth) refers to the use of mobile devices, mobile phones, and wireless technologies to support the achievement of health objectives [1]. mHealth is expected to improve access to care, enhance patient satisfaction, and reduce clinic visits and admissions without compromising safety of care and is argued to improve interaction with and participation of better-informed and more active patients [2-4]. To date, mHealth has mostly focused on patients with chronic conditions or healthy individuals to improve healthy lifestyle habits [5-7]. As in other domains of health care, including pregnancy care, a shift is currently occurring from hospital-based to home-based services [8]. In search of improved care for pregnant women, tailored care with the integration of mHealth has been suggested as an addition to or partial replacement of frequent prenatal visits [9]. This approach is called blended care, where digital technologies are combined and integrated with face-to-face care. While many of these technologies are being developed and implemented, little is still known about clinical outcomes including safety, effectiveness, patient satisfaction, and ethical considerations.

Hypertensive disorders of pregnancy (HDP) are a primary cause of adverse maternal and neonatal outcomes worldwide and occur in 10% of pregnancies [10]. Risk groups for hypertensive complications include women with chronic hypertension, diabetes, obesity, renal disease, cardiac disease, and preeclampsia in a prior pregnancy. The proportion of women with these risk factors has been steadily rising over recent years [10]. For women considered to be at risk, guidelines recommend frequent observation of the fetal condition and the pregnant woman’s blood pressure and signs of preeclampsia [11]. Planned and unplanned visits can range from every 2 weeks to 4 times a week or even daily. The burden of these recurrent visits is significant, for both patients and their spouses and family, as well as for health care services. However, the incidence of preeclampsia with severe features is approximately 3% [12], meaning that a substantial number of monitored women, while at risk, do not develop this condition.

Given the wide ubiquity of smartphones and tablets in most countries, mHealth is a promising alternative for monitoring hypertension during pregnancy. The latest research has shown that pregnant women are willing to undertake repeated self-measurements and a majority of women would like to be involved in their blood pressure management [13,14] and regard remote monitoring important for their pregnancy follow-up [15]. Little is known about the experiences of women using such platforms and how these digital tools can be aligned with their needs and preferences.

This study aimed, firstly, to explore how pregnant women, who have used mHealth as part of a blended care approach for repeated blood pressure measurements and preeclampsia symptom reporting, experience the use of such technology. Second, the study aimed to formulate recommendations based on these user experiences. Based on the insights originating from the users’ experiences, we identified several recommendations to design and implement similar interventions and to enhance future use of digital technology in clinical care.

## Methods

A mixed methods study, alongside a prospective blended care study (SAFE@home study) [16,17], was performed to explore the understanding of patients’ experiences with mHealth [18,19]. Data were collected by means of validated questionnaires and semistructured in-depth interviews with patients that had experience with mHealth for remote monitoring of HDP, to explore their experiences and motivations. The research ethics committee of the University Medical Center Utrecht determined that this study was exempt from the Medical Research Involving Humans Act (reference number 18-898-C).

### Context of the Blended Care Approach in Prenatal Care

**Figure 1:**
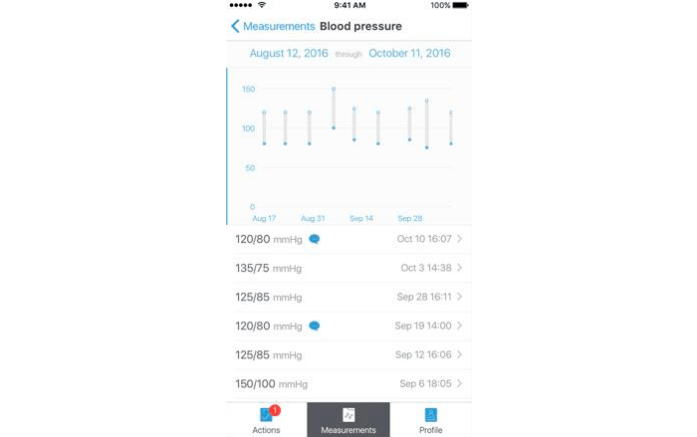
Example of the blood pressure trend graph in the Luscii app.

The overarching prospective study, named Safe@Home, evaluates the use of mHealth technology to remotely monitor blood pressure and preeclampsia symptoms. The data collected within this study were sent by the patient to the digital monitoring team, who reviewed the data each day except for the weekend days. The mHealth technology consisted of an automated blood pressure monitor with Bluetooth connection to a smartphone app for iOS users and a web-based portal for Android users (Figure 1) [16,17]. Digital monitoring started from 16 weeks gestational age and was continued until delivery, with interruption in case of hospital admission**.** Participation in the blended care approach was offered to pregnant women whom, at intake, presented with one of the following risk factors for hypertensive complications: chronic hypertension, history of prior preeclampsia, or maternal cardiac or renal disease requiring prenatal care in our clinic in the University Medical Centre Utrecht (university hospital) or Diakonessenhuis Utrecht (general teaching hospital). Access to a smartphone or tablet with internet connection and good understanding of either the Dutch or English language were required. More information about the overarching study can be found in [16,17].

A prenatal visit schedule was predefined for this group of patients, with a reduced number of visits while continuing remote monitoring. Participants were asked to measure their blood pressure every weekday before 10 am and at least 1 hour after waking up. A 9-question symptom score list could be answered in case of hypertension. Predetermined thresholds (systolic blood pressure >140 mm Hg, diastolic blood pressure >90 mm Hg) or self-reported symptoms of preeclampsia in the questionnaire resulted in automatically generated alarm signals on a monitoring dashboard in the hospital. For health care providers, a web portal provided online access to patient-reported questionnaires and blood pressure data. Members of the digital monitoring team (midwives or obstetric nurses) reviewed the data every morning from the outpatient department. The combination of blood pressure measurement and the presence of symptoms was reviewed and if needed, the digital monitoring team could consult the obstetrician for advice. Subsequently, participants were contacted to advise about management or follow-up. The platform was embedded into prenatal care with the use of a reduced predefined prenatal visit schedule, with regular appointments in the outpatient department carried out by hospital midwives and gynecologists (in training).

### Data Collection

#### Questionnaires

At 36 weeks of gestation, two questionnaires were sent by email to all participants of the prospective study. One questionnaire assessed the usability of the mHealth technology (via an app or web portal) and the connected devices, focusing on the ease of use and given instructions. This usability questionnaire consisted of 9 propositions rated on a 5-point Likert scale (strongly agree to strongly disagree) to obtain quantifiable scores (see Textbox 1). Furthermore, the use of the blood pressure monitor, usability of the smartphone app, and content of the app could be rated on a scale from 1 to 10. The usability questionnaire was generated by the study team and not validated before the start of the study. The second questionnaire was the validated Client-Centered Care Questionnaire (CCCQ). The CCCQ was developed as an instrument to measure client-centeredness as experienced by clients of care organizations and to evaluate the effects of interventions aimed at improving the client-centeredness of care services [20]. Themes of the CCCQ include recognition, respect, autonomy, and partnership as perceived by the participants. It consists of 15 questions rated on a 5-point Likert scale, ranging from “totally disagree” to “totally agree” (Multimedia Appendix 1). Results of the CCCQ can be interpreted using a unidimensional application. This is done by aggregating all information in one measure and calculating a total test score. This total score expresses care receivers’ perception of client-centered care, with higher scores representing higher perceived client-centered care. Separate questions are discussed thematically, in line with the qualitative results of this study.

**Items on the usability questionnaire.**

- The system and its use were easy to understand.

- I felt at ease using the system during pregnancy.

- While using the system, I was able to continue my daily activities.

- I am satisfied with the ease of use of this system.

- I would recommend this system to other pregnant women.

- The instructions for use of the blood pressure monitor were clear.

- The instructions for use of the app were clear.

- It was clear when to provide my measurements.

- It was clear when to contact my health care provider.

#### Interviews

Semistructured in-depth interviews were conducted with participants of the overarching study after an email invitation. Patients who were willing to be interviewed and were able to speak either Dutch or English were included in the interview study through purposive sampling. The topic list was designed to include the motivations, experiences, and perspectives of patients using the platform. The semistructured format provided participants with the opportunity to discuss matters they believed needed emphasis, while offering guidance throughout the interview. Questions for the interview guide were based on preliminary quantitative results from our questionnaires, which suggested the importance of technical functioning, communication with care providers, and implications for autonomy. The topic list was expanded based on the literature on ethical aspects of digital health, mHealth, and digital monitoring. Interviews were conducted by KJ (assistant professor) and MD (research assistant), both female researchers with experience in qualitative studies using interviews. No relationship with participants was established prior to the interviews. The interviews were conducted until saturation was reached, meaning that no new perspectives or themes were found in consecutive interviews and no new themes emerged from the data. Verbatim transcriptions of interviews and interviewers’ notes were compared with audio recordings to check for accuracy. Transcripts were imported into NVivo12 and analyzed thematically, combining inductive and deductive analyses. KJ and MD started with an a priori coding scheme to allow for deductive coding based on topics described in the literature (responsibilities, shared decision making, patient empowerment, motivations). Codes and their meanings were discussed among the research team prior to coding to guarantee intercoder reliability. The inductive part of the thematic analysis combined methods of close reading and constant comparison; codes emerging from the transcripts were clinical expertise, reassurance, burden and stress, and understanding one’s own condition. Codes were examined and systematically reviewed for supporting or conflicting evidence concerning emerging themes and codes. We also explored whether there were any differences between nulliparous and parous women and between women with a history of HDP and those without. Where relevant, we explicitly address these differences in the results. Results are reported using the Consolidated Criteria for Reporting Qualitative research checklist.

## Results

### Patient Characteristics

Of 103 invited participants, a total of 51 participants completed both questionnaires, and one participant only completed the CCCQ (total n=52). The interviews were conducted with 11 women (8 after delivery, 3 during pregnancy) and comprised the qualitative part of this study. All interviews (n=11) took place by phone, as preferred by the participants, and lasted between 35 minutes and 58 minutes. The majority of the interviewed women (8/11) also completed the questionnaires.

The demographic data of both groups are shown in Table 1. Obstetric characteristics of relevance to this topic are indicated for the interviewees and questionnaire participants (see Table 1).

After analysis of the data from the interviews and questionnaires, we identified 4 themes. Of these, 2 themes were related to the mHealth technology itself (themes 1 and 2), and 2 themes were related to the interaction with and use of the mHealth technology (themes 3 and 4).

**Table 1:** Patient characteristics.

Parameters		Questionnaires (n=52)	Interviews (n=11)
Maternal age (years), mean (SD)		34.40 (4.127)	34.18 (2.529)
BMI (kg/m^2^), mean (SD)		24.94 (4.62)	23.88 (2.496)
**Ethnicity, n (%)**			
	Caucasian	47 (90.4)	11 (100)
	Afro-Caribbean	0 (0)	0 (0)
	Mediterranean	3 (5.8)	0 (0)
	Other	2 (3.8)	0 (0)
**Level of education, n (%)**			
	Primary school	1 (1.9)	0 (0)
	Secondary school	4 (7.7)	0 (0)
	Middle-level applied education	14 (26.9)	3 (27.3)
	Higher-level applied education	17 (32.7)	6 (54.5)
	Scientific education (university)	13 (25.0)	2 (18.2)
	Unknown	3 (5.8)	0 (0)
Nulliparous, n (%)		19 (36.5)	2 (18.2)
**HDP^a^ prior pregnancy, n (%)**			
	None	14 (26.9)	3 (27.3)
	Chronic hypertension	1 (1.9)	1 (9.1)
	Gestational hypertension	5 (9.6)	2 (18.2)
	Preeclampsia/HELLP^b^	13 (25.0)	3 (27.3)
	Not applicable (nulliparous)	19 (36.5)	2 (18.2)
**Initial diagnosis at start of SAFE@home study, n (%)**			
	Preeclampsia in prior pregnancy	10 (19.2)	2 (18.2)
	Chronic hypertension	17 (32.7)	5 (45.5)
	Cardiac disease	17 (32.7)	3 (27.3)
	Renal disease	8 (15.4)	1 (9.1)
**HDP current pregnancy, n (%)**			
	None	23 (44.2)	2 (18.2)
	Chronic hypertension	14 (26.9)	3 (27.3)
	Gestational hypertension	6 (11.5)	2 (18.2)
	Preeclampsia	9 (17.3)	4 (36.4)

^a^HDP: hypertensive disorders of pregnancy.

^b^HELLP: hemolysis, elevated liver enzymes, low platelet count.

### Theme 1: Expectations of and Satisfaction With the mHealth Technology

#### Quantitative Analysis

Analysis of the usability questionnaire showed that almost all participants (49/51, 96%) felt comfortable using mHealth. The vast majority (45/51, 88%) would recommend it to their friends and family, especially participants who had been pregnant before (97% of multiparous vs. 74% of nulliparous women). Overall, client-centeredness of the blended care approach, based on the CCCQ, was rated at an average 57.5 of 75 points (range 36-75 points), which translates to a score of 77 from a possible total score of 100. This total CCCQ score was comparable between nulliparous women (score of 76, n=19) and parous women (score of 77, n=33). Of all parous women, women with prior HDP (19/33) scored the CCCQ slightly higher (score of 79/100) than those without experience with HDP (14/51; score of 75/100).

#### Qualitative Analysis

In order to understand what is important for mHealth users, we asked the interview participants what their expectations were before they started using the digital technology in the Safe@Home study and whether their expectations were met. The most often mentioned motivations to start using the technology were the expected reassurance of being closely monitored by a health care professional (9/11; 2 nulliparous and 7 parous), better pregnancy outcomes (6/11; 3 with a history of hypertension and 3 with a history of HPD), and the prospect of fewer hospital visits (5/11; none with a history of HPD). This aligned well with their experiences; most interview participants (8/11) reported they felt reassured and safe because of the close monitoring by their obstetric care professional. The use of mHealth reduced the frequency of visits, which contributed to the users’ wellbeing and a more relaxed pregnancy experience (9/11; 2 nulliparous and 7 parous). The blended care approach also enabled timely preventative measures or interventions, which resulted in early detection of abnormalities or risks (2/11). All interview participants considered it a benefit to be able to measure their own blood pressure, especially when they experienced symptoms associated with preeclampsia. Also, when their measurement indicated normal blood pressure, the digital monitoring was considered useful and reassuring, because it would indicate that the symptoms were not caused by hypertension. Comparable to the results of the questionnaire, all interviewed women would recommend the system to other pregnant women.

Some reflections of the interview participants indicated that their expectations did not always match their experiences. A few women were surprised by health care professionals calling when they did not expect it, while at other times, they were not called by the health care professional when they expected it based on their uploaded blood pressure data (2/11; both with a history of hypertension). Participants who needed reassurance that their blood pressure or symptoms were nothing to worry about sometimes called the hospital themselves. Furthermore, one interview participant needed several extra hospital visits because of hard-to-control hypertension, eventually leading to hospital admission. As a result, she was somewhat disappointed that the digital monitoring platform did not live up to her expectations (P5, Table 2).

**Table 2:** Quotes illustrating interviewees’ expectations and satisfaction.

Topic	Quotes
Reassurance	More relaxed, I’d say. I haven’t worried at all about my blood pressure. I considered it under control […] Because you do it continuously [the measurements], it reassures you. (P1)
	It is very pleasant and extremely easy. It’s reassuring that you are being monitored [by health care professionals]. (P11)
Frequency of visits	It has given me peace of mind over all those months, primarily because of the significantly reduced number of clinical visits. (P1)
	It is ironic; we expected it because it was announced like that, that we would have to visit the hospital less often, because we would be monitored via the app, but it resulted in more frequent contact. (P5)

### Theme 2: Usability of the mHealth Tool

#### Quantitative Analysis

**Figure 2:**
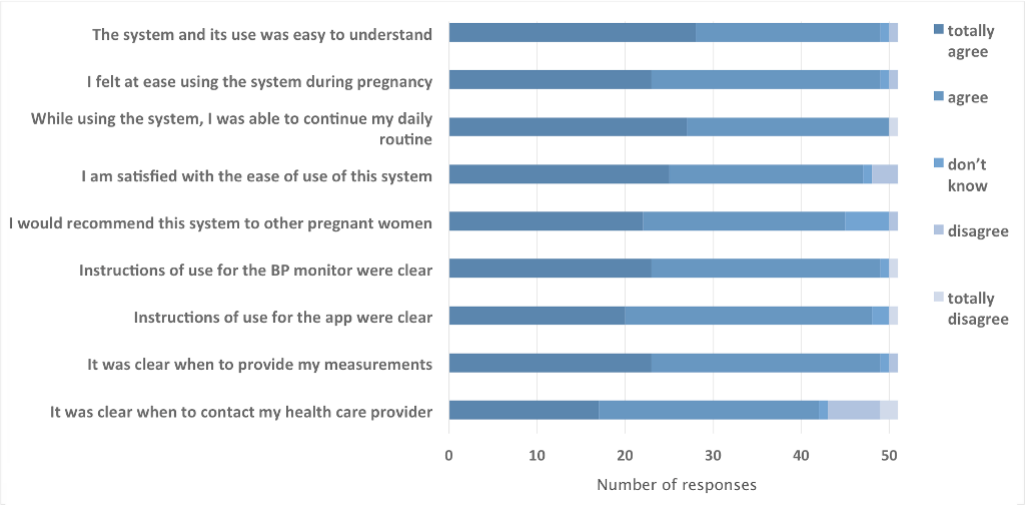
Results from the user satisfaction questionnaire.

Analysis of the questionnaires showed that nearly all participants considered the user instructions of the blood pressure monitor (49/51, 96%) and smartphone app or website (48/51, 94%) to be clear and understandable. Similarly, almost everyone (49/51, 96%) found it easy to learn how to use the mHealth technology (Figure 2).

Furthermore, the vast majority of participants (47/51, 92%) was satisfied with the usability of the mHealth technology; 81% (41/51) of the participants said the daily measurements took ≤5 minutes a day (average 4.57 minutes, range 3-15 minutes), and women could easily continue their daily routine while using the technology (50/51, 98%). Some found it difficult to combine digital monitoring with their daily routine (5/51, 10%).

On a scale from 1 to 10, the blood pressure monitor was rated at 8.5 (range 6-10), usage of the smartphone app at 7.6 (range 1-10), and content of the smartphone app at 7.8 (range 1-10).

#### Qualitative Analysis

Similar to the questionnaire, interview participants (6/11) considered the app to be “modern” and easy to use; all users of the web portal (4/11) suggested that an Android app would be more user-friendly. Moreover, the iOS app was regarded to be comprehensive; the symptom survey was considered short but clear and easy to complete — it did not take them much effort and time (4/11; 2 with a history of HPD and 2 without). Other technical aspects that contributed to the ease of use were the reminder function, automatic Bluetooth synchronization, and perceived high accuracy of the measurement. A couple of participants (2/11) noted that technical understanding of the functioning of the app was irrelevant for their user experience.

A few users mentioned that measuring early in the morning was not always easy to combine with either commuting to work or “family rush hour” in the morning (3/11) or not representative, as their morning blood pressure was naturally low (1/11). These users preferred to have the option to measure in the evening instead of the morning. Most considered measuring 5 times a week sufficient; a couple of interview participants measured every day, even during weekends, either because of worries about her medical condition (1/11; with a history of HPD) or to allow it to become a habit in their daily routine (2/11). Multiple mHealth users (4/11) measured several times a day when they experienced symptoms of preeclampsia or hypertension. At the same time, others (6/11; 4 with a history of HDP) mentioned that daily measurements were too burdensome or medicalizing, especially when they perceived their symptoms or blood pressure to be stable. A couple of interviewees (2/11) mentioned they missed the mHealth tool after giving birth and would have wanted to continue to measure during their postpartum period.

A couple of women (2/11) mentioned technical errors in the synchronization of their measurements with the system used in the hospital. Furthermore, a couple of others (2/11; both parous and with a history of hypertension) felt that the symptom score list to monitor preeclampsia signs was at times confusing because some questions did not match the specific pregnancy term. In particular, the question “Can you feel the baby move?” was considered to be upsetting in the first trimester. Also, one interview participants considered the orange or red lights stressful, as she never saw a green light because of her high values (P9, with a history of HPD; Table 3).

**Table 3:** Quotes illustrating interviewee perspectives on the usability of the mHealth tool.

Topic	Quote
App vs web portal	At first, I used the web portal, but when I had a closer look, I realized that the app is much easier, because it automatically synchronizes. It is so easy! (P1)
Frequency of measurement	Before I started, I thought it would be burdensome to measure my blood pressure every day and was not convinced that it would be necessary. [...] But eventually, it was very easy. It became part of my routine to measure my blood pressure in the morning before going to work or before bringing the children to school. (P8)
	The app was meant to be used in the morning, which was somewhat a downside, because my blood pressure is fine in the morning. (P3)
	While I was using it, no [I did not experience anything unexpected], but after giving birth and being back home, I continued measuring with my own device, because I missed that sort of information about my body. (P7)
Questions suitable to term	Those questions did not really match with being in the first trimester. Because it asked for example “do you feel contractions,” “do you still feel the baby move,” But [at that time], I hardly had a belly, and I couldn’t even feel the baby yet. [..] I found it difficult and puzzling. (P10)
Alarms	“Those lights [on the blood pressure device], they should get rid of in favor of people who are easily stressed out. They should rather show you a green light when you’re fine, orange when there are problems, and red when things are bad. [..] It showed orange so often. Since my blood pressure has been high my whole life, you feel like there is a continuous alarm, while yeah, that was not really the case.” (P9)

### Theme 3: Autonomy and Responsibility of Patients

#### Quantitative Analysis

Respondents of the questionnaires were positive about their role within the blended care approach. The majority of the participants felt they were given sufficient opportunity to draw on their own knowledge and experience (40/51, 77%) and to decide about the kind of care they receive (43/52, 83%). Furthermore, they felt they were given enough opportunity to do what they were capable of doing themselves (47/52, 90%). However, only half of the participants (26/52, 50%) felt like they were given enough opportunity to arrange and organize prenatal care themselves. Some (30/52, 57%) would like even more influence in clinical decision making and felt that health care professionals are sometimes too quick to deny a possibility. A minority of the participants (21/52, 40%) felt like they had a say in deciding when the care was provided.

#### Qualitative Analysis

Interview participants noted two dimensions related to patient autonomy. First, all interview participants (11/11) mentioned that mHealth helped to be informed about HDP. Insights on blood pressure over time, as displayed in a trend line in their app, was especially considered to be informative (6/11; all parous). Such information raised awareness about the symptoms of HDP and when to report to health care professionals (4/11). Some interview participants (2/11) argued that these insights are paired with responsibilities to carefully measure blood pressure and to contact the health care professional when symptoms increase.

A second aspect related to patient autonomy mentioned by participants was that the use of mHealth contributed to them being in control of their own health and to bring their own perspectives to the fore in consultations with health care professionals (7/11). mHealth allowed them to monitor their own symptoms and, when necessary, adapt their behavior (4/11), for example with regard to activities or medication (P8, Table 4).

**Table 4:** Quotes illustrating interviewee perspectives on autonomy and responsibilities of patients.

Topic	Quote
Being informed	I experienced that I thought I was going to measure hypertension because I felt a headache, but then I didn’t measure anything abnormal. That is odd. But exactly because of such experiences, I consider it beneficial to be able to measure, because it provided objective information to really judge it. Because I find it difficult to determine what is the matter, simply by how I feel. (P8)
	I then understood, you know, why they [health care professionals] ask you all these questions and that these are relevant. Because of the symptom score list or due to hypertension and related symptoms of preeclampsia, that I became aware that once you experience such symptoms, you shouldn’t think it’s normal, but that you have to inform health care professionals. (P2)
Information for lifestyle	It is information, you know. I had to take care of my activity level; when I did not measure hypertension, I would go outside for example. […]. And in case I would measure hypertension, I would take it easy and ask my husband to take care of the children. It provides information relevant for such activities. That was very helpful. (P8)
Responsibilities	[..] but it is also your own responsibility, the responsibility of the mother or the pregnant woman. Not only because you know your body best, but also because you become aware of aspects because of this research study. And then it’s my responsibility to discuss it with the health care professional. (P2)
Control	You both have access to the information. What I see in my overview, the physician can also see, so you can also look at it together. I got the impression that more deliberation is possible, that you do it together like how should I interpret this and the physician can explain it for example. (P8)

### Theme 4: Health Care Professionals’ Expertise and Responsibilities

#### Quantitative Analysis

After starting the digital monitoring, it was clear for the majority of the participants when the digital monitoring team needed to receive their measurements (49/51, 96%) and when to contact the physician regardless of their data (42/51, 82%). They felt that their personal wishes were sufficiently considered by the health care professionals (46/52, 89%). Most of the survey respondents said they could tell that their obstetric care professional really listened to them (50/52, 96%) and that they were given enough opportunity to say what kind of care they needed (47/52, 90%).

#### Qualitative Analysis

In addition to the findings of the quantitative research, interviewees showed that they consider the expertise of the health care professionals important in monitoring HDP. All interview participants (11/11) said that health care professionals have invaluable clinical expertise to oversee the implications of the measurements, as well as to decide the need for additional tests, the interval of clinical visits, and medication or hospitalization. The follow-up initiated by health care professionals — either by phone or via clinical visits — contributed to the feeling of being well taken care of and met the interviewees’ expectations regarding responsibilities (5/11). Patients also mentioned that it should remain the health care professionals’ responsibility to undertake action when the measurements deviate from the norm (5/11). They felt relieved that monitoring and resulting action are not solely the patient’s responsibility (11/11).

Moreover, patients were appreciative that health care professionals acted if a patient would underestimate the severity of their situation (3/11). Patients argued that important decisions about their condition cannot exclusively be based on the information from the tool, but an expert’s clinical view is required for interpretation and to make personalized treatment decisions (5/11; Table 5).

**Table 5:** Quotes illustrating interviewees’ perspectives on expertise and responsibilities of health care professionals.

Topic	Quote
Medical expertise	Well, for me, those data, I’m not trained as a health care professional, to interpret my data. I, myself, had the [possibility] to see how my blood pressure developed over time. But the idea that health care professionals see my data and can interpret it and can ask you to come to the hospital when necessary, that is comforting. (P2)
	[..] and that they can interpret it. Like for me, it was the case that it [blood pressure] was higher than 90, even if 90 is the threshold value for me, but [they explained] that for me, you see sometimes other things happening. Then I know that, you know, it’s very helpful when a physician helps me and interprets the data. I mean, that they don’t simply tell and stick to the threshold values, but also interpret it in your specific situation. I believe the shared effort lies in me conducting the measurements and supplying that information. (P8)
	[..] it’s a shared effort. But I think the physician is leading because they know best. I mean, I know more or less which medication I take now and what sort of effect it has on me. But I don’t know, for example, whether or when I can stop taking medications and what consequences it would have; I haven’t studied for that. (P4)
Active monitoring	I really like having been called after [by a nurse], because then you have confirmation that they will undertake action when it is necessary. [..] I think it was great, that in that way also really something is done with the data you collect every day. (P7)

## Discussion

This study analyzed user experiences with a blended care approach for the monitoring of HDP (Safe@Home study). Overall, the results of the questionnaires and interviews corresponded and were supplementary. The effects of using mHealth met the expectations of the participants, who were overall very satisfied with the easy-to-use technology. mHealth was considered to support patient autonomy by providing information and ways to be in control, but the interpretation of the measurements requires the involvement of health care professionals. Participants also noted a few possibilities for improvement. With the focus on future development and implementation of mHealth in care, we extracted multiple recommendations from our results (see Textbox 2).

**Recommendations for the future use and implementation of mHealth technologies in clinical care.**

1. Be modest in the communication regarding expected group benefits of the digital health technology to prevent disappointing individual patients who do not experience these specific benefits.
2. Provide the user insight into the data; in particular, a graphic representation over time is a helpful method to foster patient knowledge and can support patients to participate in clinical decision making.
3. The mHealth data should be integrated in (electronic) health records and should be accessible to all health care professionals that are engaged in care.
4. The health care professionals should remain responsible for the interpretation of data obtained via digital monitoring, as the clinical expertise of health care professionals is necessary for the early detection of abnormalities and clinical decision making.
5. Health care professionals should be aware of (pregnant) patients’ willingness and capability to self-measure their blood pressure at home.
6. Symptom score lists and blood pressure thresholds should be personalized, meaning that the questions should be adapted to the pregnancy term and thresholds should be set to fit the user’s situation.
7. The moment and frequency of measurement should be communicated clearly but should also be sensitive and adaptable to the daily life of the user.

### Our Findings in Context

Currently, several digital technologies are being developed that moderate or replace traditional clinical care. The study described here is an excellent example of such digital health technology in the clinical context that replaces some of the care traditionally provided in the clinical setting with digital monitoring at home. Our study confirmed several findings described by other digital monitoring studies. Some comparable studies have reported on remote blood pressure monitoring in pregnancy, without in-clinic monitoring by care professionals [21,22]. For a comparable intervention with clinical monitoring, only survey data were reported [15]. Our study confirmed that pregnant women at risk of HDP are willing to participate in self-monitoring services and are capable of bearing the responsibilities of measuring their own blood pressure [15,21,22]. Our study confirmed that women who experienced HDP in a prior pregnancy, in particular, were strongly in favor of blended care approaches in prenatal care [22]. A comparable intervention for pregnant women with hypertension that included remote monitoring of blood pressure and monitoring by health care professionals reported that 83% of the participants experienced a feeling of safety and that 68% preferred to be contacted within 12 hours after the measurement in case of abnormal measurements, preferably by their midwife or obstetrician [15]. Our study found comparable feelings of appreciation and safety among the users, partly because of the follow-up by health care professionals by phone or via clinical visits. Self-measuring was found to be reassuring; when abnormal values were detected as women took and interpreted their own measurement, it was clear for the participants when to contact the clinic [21]. Other studies have also found that women prefer that blood pressure monitoring should not stop at the delivery date, but should be available postnatally, which was also expressed by our interview participants [21,22].

### Opportunities for and Challenges With Blended Care Approaches in Clinical Care

With the rapid development and implementation of digital technologies in health care settings, the need for ethical guidance and practical recommendations for the implementation of such technologies, including mHealth, is widely acknowledged by patients, health care professionals, and influential advisory councils [23-25]. With the implementation of these technologies, it becomes possible to move beyond mere speculative debates about the opportunities and challenges of mHealth and to investigate how the practice is developing. Our study explored both user experiences and the expectations of users prior to using mHealth tools for digital monitoring. User experiences depend not only on the quality of the technology but also on the expectations one has before using it. Investigating both expectations and experiences is helpful, not only to understand what may motivate pregnant women to use such technologies but also to assess whether these tools live up to users’ expectations. Our study provides several insights in that respect: less frequent hospital visits and better-informed patients were often mentioned as factors contributing to the satisfaction with this technology. This shows that some of the widely discussed promises of mHealth were met in our study. Other claims about mHealth, such as increased accessibility, cost-effectiveness, and more empowered patients [1,2], were not (fully) substantiated by our study.

Furthermore, our study indicates that ethical guidance for the use of digital technologies in health care settings differs in significant ways from concerns about digital health consumers. Using digital technologies, including mHealth, in health care settings raises a wider range of ethical challenges than have been described in the consumer context [26,27]. Aside from concerns about effectiveness, privacy, and safety, the health care context requires us to carefully assess the delegation of responsibilities to patients, influence on patient autonomy, and proportionality of burden and benefits. Regarding the delegation of responsibilities, our study showed that users are able to bear the responsibility for measuring their own blood pressure, but they did not feel able to bear the responsibility of interpreting their own data. Clinicians play an important role in the responsible use and implementation of these technologies. This indicates that careful consideration is required regarding which tasks and responsibilities can be delegated to technology (instead of face-to-face care) and which can be delegated to patients (instead of the health care professionals) without compromising safety or quality of care. Digital technologies, including mHealth, are not a stand-alone solution in the clinical context and need to be supplemented with clinical expertise. With regard to the influence on patient autonomy, our study has supported evidence that patients can become more familiar with their own body and disease symptoms and are able to use this information in adjusting their behavior or to deliberate with physicians. It is important to recognize that supporting and respecting patient autonomy are not completely in their own hands. Health care professionals involved in blended care play a crucial role. Not only will health care professionals have to recognize and respect wishes of autonomous persons but will also have to navigate between the standardized way of measuring, supported by digital technology, while still being able to personalize the analysis and interpretations to the interests and needs of a specific patient. Lastly, while mHealth technologies have several benefits, such as accessibility of information for both patients and health care professionals, less frequent hospital visits, and better understanding of one’s own conditions, these benefits need to outweigh the burden of using these technologies (eg, time investment, user friendliness). Overall, our participants were very positive and satisfied with the mHealth technology, but the interview participants who felt their blood pressure was stable because of prescribed medication argued that the burden of measuring every day became somewhat disproportionate. Less frequent measurements may be a way to balance the burden and benefits for these groups. It also indicates that high levels of satisfaction with this blended care approach might be specific to the high-risk population that was selected for this approach. For the high-risk population, there is much to gain in terms of both health outcomes and time investment, but the balance may tip differently for medium-risk to low-risk groups.

### Strengths and Limitations

This is a mixed methods study that benefits from reducing weaknesses inherent to both methods; it expands understanding, while also being comprehensive. Approximately half of the total users of the mHealth technology filled out the validated questionnaires. The sample of interviewees was representative of the participants of the questionnaires in terms of age, BMI, education level, and underlying conditions (Table 1). The findings of the survey and interviews were supplementary and helped to better understand what and for which reasons the mHealth tool was appreciated, which can inform future mHealth health interventions.

Our results must be interpreted in the context of the following limitations. Selection bias (self-selection) might have influenced the results, as participants of the prospective study agreed to take part in this innovative strategy with digital monitoring and may thus have a positive attitude in general to mHealth. Furthermore, the women willing to participate in this study may have had a relatively positive experience with this specific technology. Also, the experience of participants could have been biased by the outcome of their pregnancy. However, as the findings of the questionnaires (collected during pregnancy) and the interview data (8 postpartum, 3 during pregnancy) correspond, the influence may be marginal. The interviewed patients were fairly highly educated and may therefore not be representative of pregnant women in other socioeconomic situations. This explorative study has a relatively small sample size; in both the quantitative and qualitative aspects of the study, the provided ratios and percentages were not statistically powered and therefore cannot be fully generalized to other populations or care settings. Although saturation was reached on the identified codes and themes, further research could investigate these topics in more depth.

### Conclusions and Recommendations

Our study explored the perspectives of pregnant women regarding the use of mHealth in a blended care approach to remotely monitor blood pressure in pregnancy. Based on the experiences of the users, several recommendations have been formulated. These recommendations draw on the needs, experiences, and views of the patients, meaning that following these recommendations will contribute to better-aligned and patient-centered care. These recommendations can help other scholars or physicians to guide the process of implementation and design of similar mHealth technologies.

## Appendix

Multimedia Appendix 1 Client-centered-care questionnaire (CCCQ).

## Abbreviations

CCCQ: Client-Centered Care Questionnaire
HDP: hypertensive disorders of pregnancy
mHealth: mobile health
