# Supplementary material for: User Experiences With and Recommendations for Mobile Health Technology for Hypertensive Disorders of Pregnancy: Mixed Methods Study
Source: JMIR Mhealth Uhealth. 2020 Aug 4;8(8):e17271. doi: 10.2196/17271 (PMC7435610; doi:10.2196/17271)
Supplement: Multimedia Appendix 1 [file mhealth_v8i8e17271_app1.pdf]

Appendix 3 : – client-centered-care questionnaire (CCCQ)

1.1 I can tell that the carers take my personal wishes into account.

Item		Participants n=52	
1 – strongly disagree	Number (%)	0 (0%)	
2 – disagree	Number (%)	0 (0%)	
3 – neither disagree nor agree	Number (%)	6 (11.5%)	
4 – agree	Number (%)	34 (65.4%)	46 (88,5%)
5 - strongly agree	Number (%)	12 (23.1%)	

1.2 I can tell that the carers really listen to me.

Item		Participants n=52	
1 – strongly disagree	Number (%)	2 (3.8%)	
2 – disagree	Number (%)	0 (0%)	
3 – neither disagree nor agree	Number (%)	0 (0%)	
4 – agree	Number (%)	38 (73.1%)	50 (96,1%)
5 - strongly agree	Number (%)	12 (23.1%)	

1.3 I can tell that the carers take into account what I tell them.

Item		Participants n=52	
1 – strongly disagree	Number (%)	1 (1.9%)	
2 – disagree	Number (%)	3 (5.8%)	
3 – neither disagree nor agree	Number (%)	1 (1.9%)	
4 – agree	Number (%)	33 (64.5%)	47 (90,4%)
5 - strongly agree	Number (%)	14 (26.9%)	

1.4 I get enough opportuniy to say what kind of care I need.

Item		Participants n=52	
1 – strongly disagree	Number (%)	0 (0%)	
2 – disagree	Number (%)	1 (1.9%)	
3 – neither disagree nor agree	Number (%)	4 (7.7%)	
4 – agree	Number (%)	32 (61,5%)	47 (90,4%)
5 - strongly agree	Number (%)	15 (28.8%)	

1.5 I can tell that the carers respect my decision even though I disagree with them.

Item		Participants n=52

1 – strongly disagree	Number (%)	0 (0%)	
2 – disagree	Number (%)	0 (0%)	
3 – neither disagree nor agree	Number (%)	22 (42.3%)	
4 – agree	Number (%)	22 (42.3%)	30 (57,7%)
5 - strongly agree	Number (%)	8 (15.4%)	

1.6 In my opinion the carers are clear about what they are able and allowed to provide.

Item		Participants n=52	
1 – strongly disagree	Number (%)	0 (0%)	
2 – disagree	Number (%)	1 (1.9%)	
3 – neither disagree nor agree	Number (%)	7 (13.5%)	
4 – agree	Number (%)	30 (57.7%)	44 (84,6%)
5 - strongly agree	Number (%)	14 (26.9%)	

1.7 In my opinion the carers are sometimes too quick to say that something is not possible.

Item		Participants n=52	
1 – strongly agree	Number (%)	1 (1.9%)	
2 – agree	Number (%)	6 (11.5%)	
3 – neither disagree nor agree	Number (%)	15 (28.8%)	
4 – disagree	Number (%)	20 (38.5%)	30 (57,7%)
5 - strongly disagree	Number (%)	10 (19.2%)	

1.8 I'm given enough opportunity to use my own expertise and experience with respect to the care I need.

Item		Participants n=52	
1 – strongly disagree	Number (%)	0 (0%)	
2 – disagree	Number (%)	2 (3.8%)	
3 – neither disagree nor agree	Number (%)	10 (19.2%)	
4 – agree	Number (%)	28 (53.8%)	40 (76,9%)
5 - strongly agree	Number (%)	12 (23.1%)	

1.9 I'm given enough opportunity to do what I am capable of doing myself.

Item		Participants n=52	
1 – strongly disagree	Number (%)	0 (0%)	
2 – disagree	Number (%)	0 (0%)	
3 – neither disagree nor agree	Number (%)	5 (9.6%)	
4 – agree	Number (%)	38 (73.1%)	47 (90,4%)
5 - strongly agree	Number (%)	9 (17.3%)	

1.10 I'm given enough opportunity to help decide on the kind of care I receive.

Item		Participants n=52	
1 – strongly disagree	Number (%)	0 (%)	
2 – disagree	Number (%)	1 (1.9%)	
3 – neither disagree nor agree	Number (%)	8 (15.4%)	
4 – agree	Number (%)	31 (59.6%)	43 (82,7%)
5 - strongly agree	Number (%)	12 (23.1%)	

1.11 I'm given enough opportunity to help decide on how often I receive care.

Item		Participants n=52	
1 – strongly disagree	Number (%)	1 (1.9%)	
2 – disagree	Number (%)	2 (3.8%)	
3 – neither disagree nor agree	Number (%)	15 (28.8%)	
4 – agree	Number (%)	22 (42.3%)	34 (65,4%)
5 - strongly agree	Number (%)	12 (23.1%)	

1.12 I'm given enough opportunity to help decide on how the care is given

Item		Participants n=52	
1 – strongly disagree	Number (%)	1 (1.9%)	
2 – disagree	Number (%)	4 (7.7%)	
3 – neither disagree nor agree	Number (%)	13 (25.0%)	
4 – agree	Number (%)	25 (48.1%)	34 (65,4%)
5 - strongly agree	Number (%)	9 (17.3%)	

1.13 I have a say in deciding on when carers come to help me.

		Participants n=52	
1 – strongly disagree	Number (%)	3 (5.8%)	
2 – disagree	Number (%)	11 (21.2%)	
3 – neither disagree nor agree	Number (%)	17 (32.7%)	
4 – agree	Number (%)	17 (32.7%)	21 (40,4%)
5 - strongly agree	Number (%)	4 (7.7%)	

1.14 In my opinion, I am consulted sufficiently on who provides the care.

Item		Participants n=52	
1 – strongly disagree	Number (%)	1 (1.9%)	
2 – disagree	Number (%)	9 (17.3%)	
3 – neither disagree nor agree	Number (%)	13 (25%)	
4 – agree	Number (%)	24 (46.2%)	

5 - strongly agree	Number (%)	5 (9.6%)	29 (55,8%)

1.15 I'm given enough opportunity to arrange and organize the care provided myself.

<table>
<tr><td>Item</td><td></td><td colspan="2">Participants n=52</td></tr>
<tr><td>1 – strongly disagree</td><td>Number (%)</td><td colspan="2">1 (1.9%)</td></tr>
<tr><td>2 – disagree</td><td>Number (%)</td><td colspan="2">8 (15.4%)</td></tr>
<tr><td>3 – neither disagree nor agree</td><td>Number (%)</td><td colspan="2">17 (32.7%)</td></tr>
<tr><td>4 – agree</td><td>Number (%)</td><td>23 (44.2%)</td><td rowspan="2">26 (50,0%)</td></tr>
<tr><td>5 - strongly agree</td><td>Number (%)</td><td>3 (5.8%)</td></tr>
</table>
